# Study and Optimal Design of the Integrated 37° Unidirectional SV-EMAT for Rapid Rail Flaw Detection

**DOI:** 10.3390/s25247424

**Published:** 2025-12-06

**Authors:** Wei Yuan

**Affiliations:** Infrastructure Inspection Research Institute, China Academy of Railway Sciences Corporation Limited, Beijing 100081, China; 18519652204@163.com; Tel.: +86-185-1965-2204

**Keywords:** electromagnetic acoustic transducer, railway detection, rapid detection, detection scan distance

## Abstract

The problem of poor coupling and wheel breakage is a critical issue in the rapid inspection of rails using contact piezoelectric ultrasonic technology for trolleys and vehicles. To overcome this shortcoming, a non-contact unidirectional Shear Vertical Wave EMAT (USV-EMAT) for rapid rail flaw detection with a larger emission angle is proposed and optimized. First, the core characteristics of the USV-EMAT and the Unidirectional Line-Focusing Shear Vertical Wave EMAT (ULSV-EMAT) are compared and analyzed, including emission angle, directivity, intensity, and detection scan distance. The results confirmed that the USV-EMAT is more suitable for rapid rail flaw detection. Secondly, the orthogonal experimental analysis method was used to optimize the structural parameters of the probe. This study systematically identified the key factors influencing the directivity and intensity of acoustic waves excited by the probe, as well as the detection blind zones. Finally, the structural parameters of the integrated 37° USV-EMAT probe were determined by comparing and analyzing the received signal characteristics of the transmit–receive racetrack coil and the self-transmitting–receiving meander coil. The results show that the optimized probe achieves a 14.3 dB SNR for detecting a 5 mm diameter, 50 mm deep transverse hole in the rail, and a 14.0 dB SNR for a 3 mm diameter, 25 mm long, 50 mm deep flat-bottomed hole. Additionally, this study reveals that as the burial depth of the transverse holes increases, the detection scan distance for such defects exhibits an “N”-shaped trend, with the minimum occurring at a depth of 90 mm.

## 1. Introduction

As a critical component of railway infrastructure, the structural health of rails directly determines the safety and stability of train operations [1,2,3]. Under prolonged cyclic loading and complex environmental conditions, minor internal defects in rails may develop into macroscopic cracks if left unaddressed, potentially triggering catastrophic failures such as rail fractures [4,5,6]. Therefore, creating an efficient and accurate rail flaw detection system is pivotal to safeguarding railway safety.

Presently, rail defect detection mainly relies on four inspection modes: handheld instruments, hand-pushed trolleys, double-rail carts, and rail flaw detection vehicles. Handheld instruments adopt a static detection approach, enabling precise rail inspections [7,8]. Nevertheless, they are constrained by manual operation, resulting in low detection efficiency, and are only applicable to localized inspections, such as those for welds and turnouts. In comparison, inspections using hand-pushed trolleys, double-rail carts, and rail flaw detection vehicles boast faster detection speeds and can undertake full-line rail flaw detection tasks [9]. All these traditional detection methods utilize piezoelectric ultrasonic probes and necessitate the use of an acoustic couplant to ensure effective transmission of acoustic energy. However, the coupling approach exhibits significant limitations in practical applications. Specifically, at low temperatures, the coupling water is prone to freezing, which disrupts acoustic coupling and severely compromises the accuracy and reliability of inspections [10].

Electromagnetic Acoustic Transducer (EMAT) technology, with its advantages of non-contact excitation, no need for a couplant, and strong environmental adaptability, provides an innovative solution for faster dynamic rail flaw detection. Many scholars have conducted in-depth research on applying this technology for rail inspection. Dixon et al. [11,12,13] utilized EMAT-excited Rayleigh waves and guided waves to detect transverse or longitudinal cracks in rail heads. They also designed a surface wave EMAT with a magnet lift-off distance increased to 10 mm, achieving a detection speed of up to 160 km/h. Mi et al. [14] designed an EMAT narrowband surface wave coil, which successfully detected various defects, such as rail cracks and nuclear damage. Lu et al. [15] effectively identified rail tread cracks and switch rail base damage using surface waves and SH-guided waves. These researchers employed low-frequency guided waves of different modes to inspect the railhead. It should be noted that as the frequency decreases, the wavelength increases, resulting in lower sensitivity to defects and a higher probability of missed detections. Additionally, both the Rayleigh wave and SH-guided wave propagate along the surface, enabling detection of only surface defects in rails. Liu et al. [16] demonstrate that rail subsurface inspection by using a spiral-coil EMAT is feasible and the high frequency can contribute to distinguishing the echo signal from the rail subsurface crack. However, according to the relevant standards for locomotive vehicle clearance in standard gauge railway requirements, ultrasonic sensors must be installed above the rail surface to avoid interference with the track during operation.

Rail defects typically originate internally and propagate at an angle. To detect more defects, probes need different incident angles. These angles include 0°, 37°, and 70°, just like traditional rail flaw detection vehicles. Liu et al. [17] optimized the design of Halbach array permanent magnets for a butterfly coil EMAT, which remarkably enhanced both signal strength and defect recognition capability. However, a notable limitation of this optimized design is that it can only generate shear waves at 0°. Tu et al. [18] proposed a composite EMAT that utilizes 30° bidirectional Shear Vertical Waves (BSV-EMAT) with a frequency of 1.59 MHz and surface waves with a frequency of 737 kHz simultaneously. This sensor enables simultaneous flaw detection on the surface and inside of the railhead. Li [19] proposed a horizontal magnetization BSV-EMAT, which achieved a 60° incidence angle of an SV wave. Notably, neither 30° nor 60° satisfies the angle requirement for rail flaw detection. Moreover, the BSV-EMAT inherently excites two symmetric main pulses centered on the sensor, which results in ambiguity regarding the echo direction during flaw detection and ultimately leads to inaccurate defect localization. Su et al. [20] designed a ULSV-EMAT capable of accurately detecting defects in the rail base center. However, its 1 MHz excitation frequency and 50° incident angle make it better suited for static detection scenarios, rendering it incompatible with dynamic inspection devices. Cai et al. [21] optimized the ULSV-EMAT parameters. They used fan-shaped scanning imaging technology and thickness step-surface recognition. This improved detection signal strength. It also raised defect recognition accuracy. Wang et al. [22] improved the EMAT probe. They developed a low-frequency SV-wave EMAT. It significantly enhanced signal strength and reduced suction with the rail. Tests have proven that it can detect rail base defects. The ULSV-EMATs investigated by these scholars exhibit significant advantages in the field of static rail defect detection, as they provide high excitation intensity and possess robust signal reception capabilities. However, their design does not fully accommodate the dynamic detection requirements of railway systems.

As railway operation mileage continues to increase, the pressure for effective rail flaw detection intensifies daily. Consequently, rapid detection has emerged as an essential trend within the industry. This study addresses the urgent need for dynamic rail detection by systematically analyzing performance parameters associated with the Unidirectional Shear Vertical Wave EMAT (USV-EMAT), and the Unidirectional Line-Focusing Shear Vertical Wave EMAT (ULSV-EMAT). Key parameters examined include emission angle, directivity, and detection scan distance. Based on this analysis, a USV-EMAT is designed specifically for rapid detection purposes. The objective is to overcome existing technical bottlenecks while enhancing both efficiency and accuracy in dynamic rail flaw detection.

## 2. SV Wave Detection Principle and Characteristics of EMAT

Rail defects are commonly categorized into two classes: volumetric defects and planar defects. Owing to their morphology, volumetric defects can generally be detected effectively with probes deployed over a broad range of incidence angles. By contrast, planar defects tend to be oriented at characteristic angles, most frequently near 37° or 70°. Their anisotropic geometry requires angle-matched insonification for reliable identification. In current practice, large-rail flaw detection vehicles and hand-pushed or double-rail flaw detection trolleys employ contact piezoelectric probe arrays with nominal angles of 0°, 37°, and 70°, providing complementary coverage across defect types and orientations. To enable non-contact inspection while maintaining angle selectivity for both volumetric defects and planar defects developing at approximately 37°, this study optimizes and designs an EMAT configured with a 37° incident angle and an excitation frequency of 2.25 MHz. The proposed configuration targets efficient coupling of SV modes required for angle-specific interrogation, thereby addressing practical constraints of field-deployable rail inspection systems.

The EMAT consists of three components: permanent magnet, excitation/reception coil, and test specimen. By adjusting the magnet coil structure, different ultrasonic wave modes can be generated. At present, there are four structures that can excite the required oblique incidence, namely Bidirectional SV EMAT (BSV-EMAT), Line-Focused SV EMAT (LFSV-EMAT), Unidirectional SV EMAT (USV-EMAT), and Unidirectional Line-Focused SV EMAT (ULFSV-EMAT). Among them, BSV-EMAT and LFSV-EMAT radiate in two opposite azimuth directions. This bidirectional transmission mode has disadvantages in on-site detection, as the echoes may come from both sides of the probe, leading to complex path allocation and introducing ambiguity in defect localization, thereby reducing detection accuracy and reliability.

**Figure 1:**
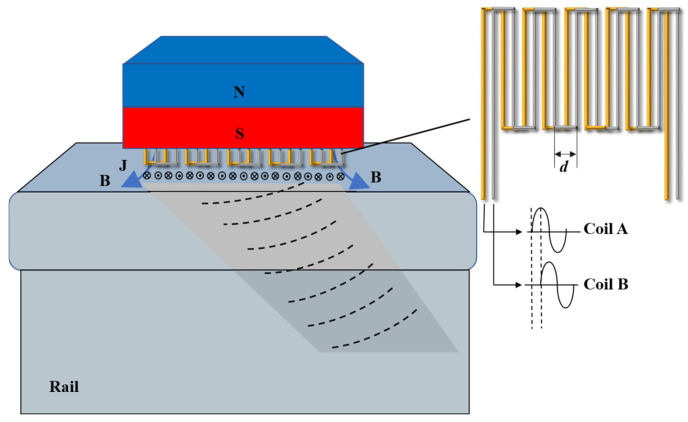
Working principal diagram of the USV-EMAT.

**Figure 2:**
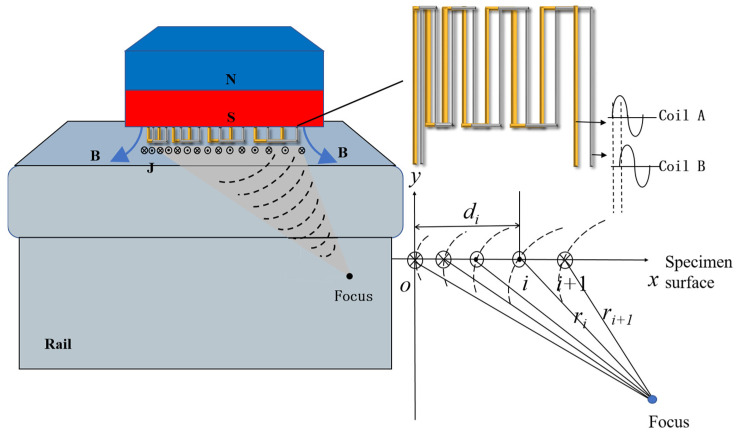
Working principal diagram of the LFSV-EMAT.

Figure 1 and Figure 2 illustrate the structural schematics of USV-EMAT and ULFSV-EMAT. As depicted in Figure 1, the USV-EMAT is classified as an oblique-incidence EMAT with a fixed coil spacing pattern; specifically, its curved coil maintains a constant spacing denoted as *d*. The primary radiation angle *θ* of the ultrasonic wave can be determined using the following equation:

(1) $$sin\;\theta =\frac{(2n\;+\;1)c}{2df}=\frac{\lambda (2n\;+\;1)}{2d}$$

In Equation (1), *f* is the frequency of the excitation signal. *θ* is the deflection angle of the ultrasonic wave beam. *n* is a positive integer. *c* is the propagation speed of the ultrasonic wave in the specimen. When the excitation frequency *f* is maintained constant, adjusting the line spacing *d* of the curved coil enables precise regulation of the directivity of the incident oblique ultrasonic beam.

The LFSV-EMAT is an oblique-incidence EMAT. It has a variable coil spacing pattern. Wire spacing is affected by multiple factors. These include focal point coordinates, incident angle, and excitation frequency. The calculation method is shown in Figure 2. Consider a set of given design parameters. They include the excitation signal frequency f. They also include the focal point coordinates ($x_{F}$, $y_{F}$). The horizontal coordinate ($x_{1}$, 0) of the leftmost coil is another. The incident angle $\theta_{1}$ concerning the focal point is also included. The position of all wires can be determined. The key relationship is this: the propagation path difference from the i-th to the (i + 1)-th source to the focal line equals half the wavelength. The calculation formula is as follows:

(2) $$r_{i}\;-\;r_{i\;+\;1}=\frac{c}{2f}$$

(3) $$d_{i}=x_{F}-\sqrt{[(x_{F}^{2}+y_{F}^{2})-\frac{c}{2f}(i-1){]}^{2}-y_{F}^{2}}$$

This study focuses on two sensors. Both have unidirectional emission characteristics. They are USV-EMAT and LFSV-EMAT. A systematic comparative analysis is conducted. It covers multiple dimensions. These include acoustic field distribution, detection sensitivity, and defect localization capability. The goal is to deliver an optimized technical solution. It is for dynamic rail inspection.

## 3. Finite Element Simulation Modeling and Analysis

### 3.1. Finite Element Simulation Modeling

**Figure 3:**
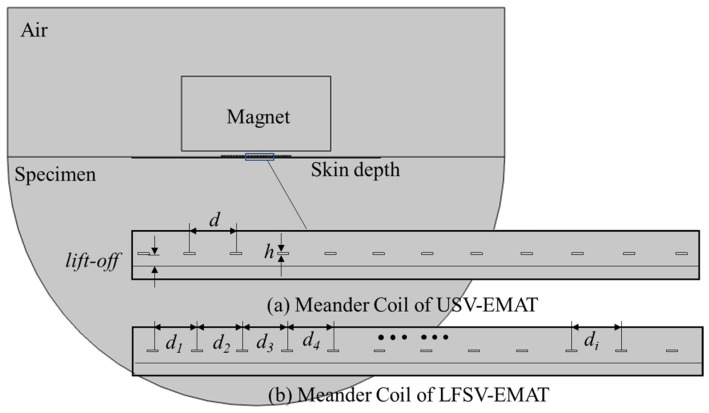
Finite element simulation modeling diagram.

Two simulation models, LFSV-EMAT and USV-EMAT, were established separately by the multi-physical field simulation software, such as COMSOL, ANSYS, etc. The model is the result of the coupling of the mechanics field and electromagnetics field. Each model includes a permanent magnet, a dual-wound meander coil, the specimen, and the surrounding air domain. To assess wavefield distribution and directivity, the specimen is modeled as a semi-circular block of radius 60 mm. Its material properties are assigned to match rail steel. The dual-wound meander coil is stacked directly beneath the magnet. As shown in Figure 3, the two models share identical parameters except for differences in coil parameters. The *d_i_* of the LFSV-EMAT coil is calculated according to Equation (1), and the *d* of the USV-EMAT coil is calculated according to Equation (3). The excitation input is applied with a frequency of 2.25 MHz, a 90° phase difference, and a peak current of 100 A, and the toneburst consists of five cycles, as described in Equations (4)–(7). To balance computational efficiency and accuracy, a mesh refinement strategy is employed for the skin layer region directly beneath the sensor and the specimen. This approach ensures the computational accuracy of the critical areas. The EMAT simulation parameters are shown in Table 1.

(4) $${f_{1}\left(t\right)=I}_{0}\left(1-cos\left(\frac{2\pi f_{0}t}{w_{r}}\right)\right)\cos\left(2\pi f_{0}t\right),\;0\leq t\leq \frac{w_{r}}{f_{0}}$$

(5) $${f_{2}\left(t\right)=I}_{0}\left(1-\cos\left(\frac{2\pi f_{0}\left(t-r\right)}{w_{r}}\right)\right)\cos\left(2\pi f_{0}\left(t-r\right)\right),\;r\leq t\leq \frac{w_{r}}{f_{0}}+r$$

(6) $$f_{3}\left(t\right)=-f_{1}\left(t\right),\;0\leq t\leq \frac{w_{r}}{f_{0}}$$

(7) $$f_{4}\left(t\right)=-f_{2}\left(t\right),\;r\leq t\leq \frac{w_{r}}{f_{0}}\;+\;r$$

### 3.2. Finite Element Simulation Analysis

**Figure 4:**
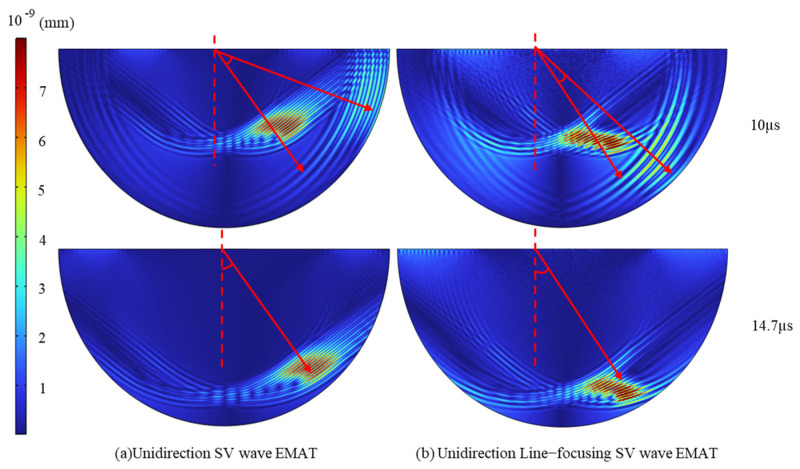
Oblique-incidence EMAT finite element simulation propagation diagram.

Based on the established USV-EMAT and LFSV-EMAT finite element simulation models of 37°, simulations are carried out to obtain the production and propagation processes of EMAT in the specimen for both structures. Figure 4a presents USV-EMAT wavefield snapshots at *t* = 10 μs and *t* = 14.7 μs, Figure 4b shows the corresponding results for LFSV-EMAT. These diagrams illustrate the behavior of the ultrasonic waves produced and propagated by the EMAT structures within the material, highlighting their performance over time at different moments.

**Figure 5:**
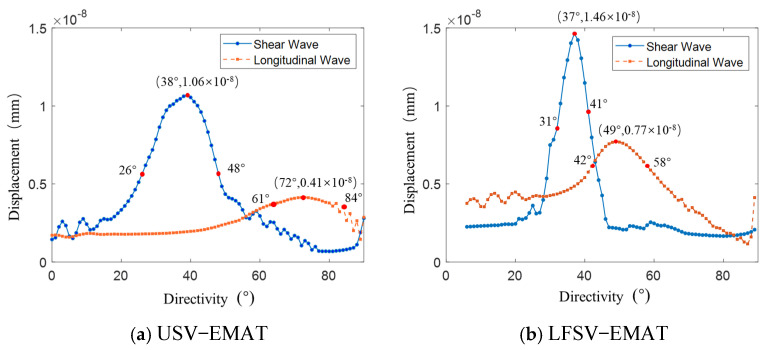
The directivity of the USV-EMAT and LFSV-EMAT.

As shown in Figure 5, both USV-EMAT and LFSV-EMAT excite SV that radiate at approximately 37°. However, their longitudinal wave (L-wave) directivity differs markedly. To quantify beam directivity, displacement signals were sampled at 1° intervals along the bottom arc of the specimen within the 0–90° central-angle range, and the corresponding polar directivity diagrams were plotted.

From the directivity data, the SV peak for USV-EMAT occurs at 38°, with a maximum amplitude of 1.06 × 10^−8^ m. The L-wave peak occurs at 72°, with a maximum amplitude of 0.41 × 10^−8^ m, resulting in a difference of 34° between the peak direction angles of SVs and L-waves. The 6 dB attenuation method, where the sound pressure amplitude decays to 1/2 of the peak value, is a standard technique in non-destructive testing used to estimate the size or boundary of a defect based on signal drop. By using it, the L-wave emission angle for USV-EMAT is calculated to be 22° (48° − 26°), and the SV emission angle is 23° (84° − 61°). For LFSV-EMAT, the SV reaches its peak at 37° with a maximum amplitude of 1.46 × 10^−8^ m, while the L-wave peaks at 49° with a maximum amplitude of 0.77 × 10^−8^ m, resulting in a 12° difference in the peak direction angles between SVs and L-waves. Applying the same 6 dB attenuation method, the L-wave emission angle is calculated to be 16° (58° − 42°), and the SV emission angle is 10° (41° − 31°). In this case, the ratio of SV to L-wave intensity is 2:1, with the L-wave emission angle being larger than that of SVs.

**Figure 6:**
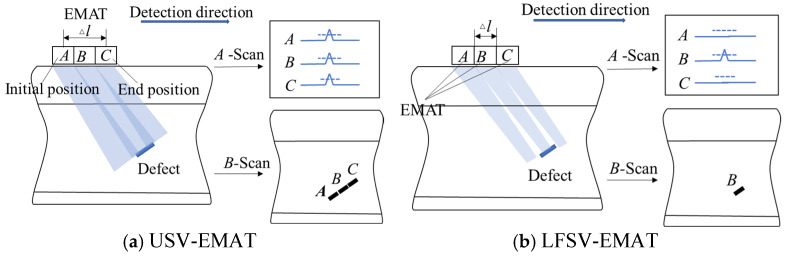
Schematic diagram of actual dynamic flaw detection scene.

The research results indicate that LFSV-EMAT has a smaller emission angle and higher acoustic wave intensity, making it more favored by researchers in conventional inspection applications. However, in dynamic inspection scenarios such as large-rail flaw detection vehicles or double-rail flaw detection trolleys, due to the detection equipment’s operational speed, the A-scan signals of the flaw can be converted into B-scan images for damage evaluation. According to the damage determination rule, the greater the number of wave emission points in the detected signal, the higher the likelihood of damage. As shown in Figure 6, when the sensor’s emission angle is larger, the same defect can be detected at multiple locations, such as A, B, and C. Defects of the same size will present more emission points and are therefore easier to identify. In summary, the distance between the initial position and the end position where the defect can be detected is referred to as the detection scan distance (Δ*l*). Although the SV intensity generated by USV-EMAT is slightly lower than that of LFSV-EMAT, its wider emission angle and longer detection scan distance (Δ*l*) make it more advantageous for application in dynamic rail inspection.

### 3.3. Optimization of Oblique Incident Probes

In oblique-incidence EMAT excitation, line spacing, number of turns, lift-off distance, coil width, and coil height are the principal design variables governing performance. To enforce a prescribed radiation angle, the phase-matching condition in Equation (1) must be satisfied; this requires a fixed line spacing. Accordingly, we select number of turns, lift-off distance, coil width, and coil height as the optimization parameters.

To reduce experimental burden and trial count, we adopt an orthogonal experimental design. The four parameters above are treated as factors, each with five levels. Let *N* denote the number of turns of the single-wire coil; the double-wire coil therefore uses 2*N* turns. The coil is fabricated on flexible printed circuit (FPC) substrates: the initial coil height equals the thickness of a single FPC layer, with additional layers stacked to reach higher levels. The factors and their levels are summarized in Table 2.

Acoustic wave directivity is a primary determinant of defect detectability. Fatigue damage in rails commonly evolves along approximately 37°; echoes generated at other angles are difficult to capture after reflection and thus contribute little to detection. Echo intensity correlates positively with the electromechanical energy conversion efficiency and the signal-to-noise ratio (SNR); higher intensity generally yields superior detection performance. Each probe exhibits a detection blind zone: defects located within this zone produce echoes masked by the initial pulse and are prone to missed detections. A smaller blind spot is therefore desirable. Consequently, we adopt directivity, echo intensity, and detection blind zone as the core metrics for evaluating probe performance. Table 2 summarizes the L25 (5^4^) orthogonal design used for simulation-driven parameter optimization, and Table 3 reports the corresponding analysis of results.

(8) $$K_{ZN}=\frac{1}{m}\sum_{i=1}^{n}\;y_{Z}i$$

(9) $$T_{Z}=R_{maxZ}-R_{minZ}$$

In the formula,

i—Sequence number;
y—Directivity, emission angle, strength, detection blind zone;
N—Level number;
$R_{maxZ}$ = max{$K_{Z1},K_{Z2},K_{Z3},K_{Z4},K_{Z5}$};
$R_{minZ}$ = min{$K_{Z1},K_{Z2},K_{Z3},K_{Z4},K_{Z5}$} (*n* = 25, *m* = 5).

In the range analysis, the mean value $K_{ZN}$ for *N* = 1, 2, 3, 4, 5 describes the influence of the Z factor on the experimental results, while the effect degree $T_{Z}$ indicates the extent of the impact of the Z factor.

**Figure 7:**
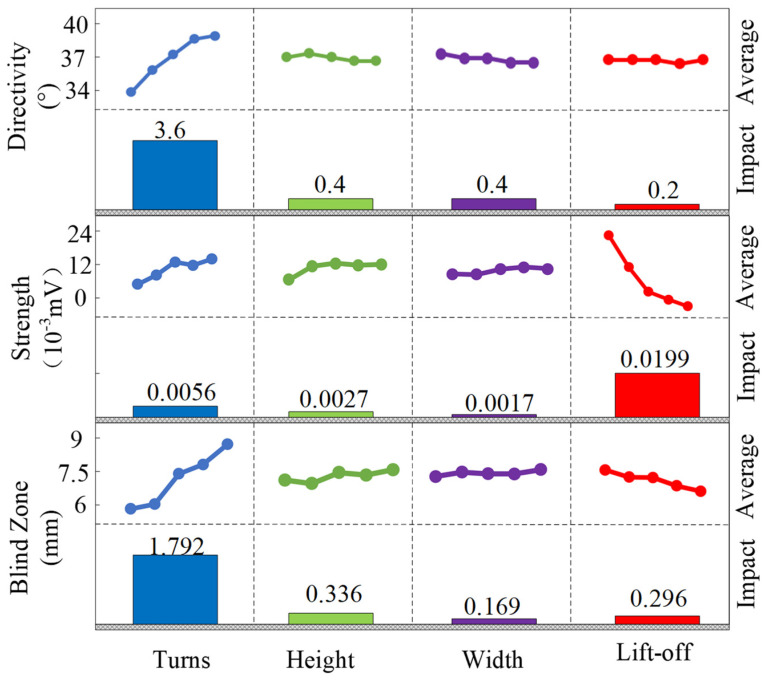
The average value and influence of each design factor on different results.

The range analysis results for different $K_{ZN}$ and $T_{Z}$ values using the above analysis method are shown in Table 4. Figure 7 presents the mean values and effect degrees of each design factor on different results. It can be observed that the most significant factor influencing acoustic wave directivity is the number of coil turns, followed by coil height and coil width, while the lift-off distance has the least impact. Acoustic wave directivity increases with the number of coil turns and slightly decreases with an increase in coil width. For coil height and lift-off distance, the directivity shows irregular changes. The best directivity of 37° is achieved when the number of coil turns is 12. Regarding echo intensity, the lift-off distance has the most significant effect, followed by the number of coil turns, coil height, and coil width, with smaller influences. Echo intensity decreases as lift-off distance increases, while it first increases, then decreases, and then increases again with the increase in the number of coil turns and coil height. As for coil width, echo intensity first decreases and then increases. For detection blind zone, the number of coil turns has the greatest impact, followed by coil height and lift-off distance, with coil width having the least effect. The detection blind zone increases as the number of coil turns increases, while it decreases as lift-off distance increases. The detection blind zone shows smaller changes with coil height and coil width. Based on the optimal parameters required, the best configuration is number of coil turns = 12, coil height = 0.04 mm, coil width = 0.4 mm, and lift-off = 0.2 mm.

### 3.4. Analysis of Received Signals of Different Coil Types

**Figure 8:**
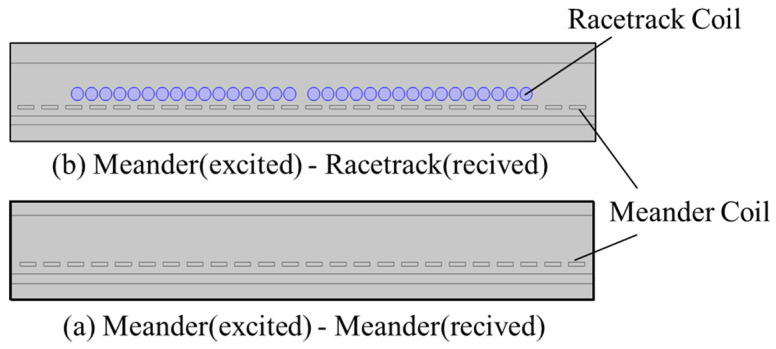
A schematic diagram of the EMAT simulation coil.

In Section 3.1, the excitation coil structure suitable for dynamic rail detection was determined through parameter optimization. Based on the principles of electromagnetic ultrasonic sensor excitation and reception, the racetrack-type coil exhibits significant advantages in bulk wave excitation and reception. To systematically explore the influence of different reception coil structures on ultrasonic wave excitation and reception performance, we use the spontaneous excitation–reception model (single meander with diplexer coil) established in Section 3.1 as the baseline for comparison. Additionally, a dual-coil model (meander excited–racetrack received) of the EMAT is constructed using a finite element model. A comparative analysis of the echo quality from different coil structures is conducted. As shown in Figure 8, compared to the spontaneous excitation–reception coil model, the one-excitation–one-reception coil model adds a racetrack received coil with 16 turns and a diameter of 0.3 mm directly above the meander coil, enabling efficient ultrasonic wave reception and signal acquisition. Certainly, a racetrack coil with a smaller wire diameter (0.1 mm) and a higher number of turns (30) was also tested, which may yield more favorable results.

**Figure 9:**
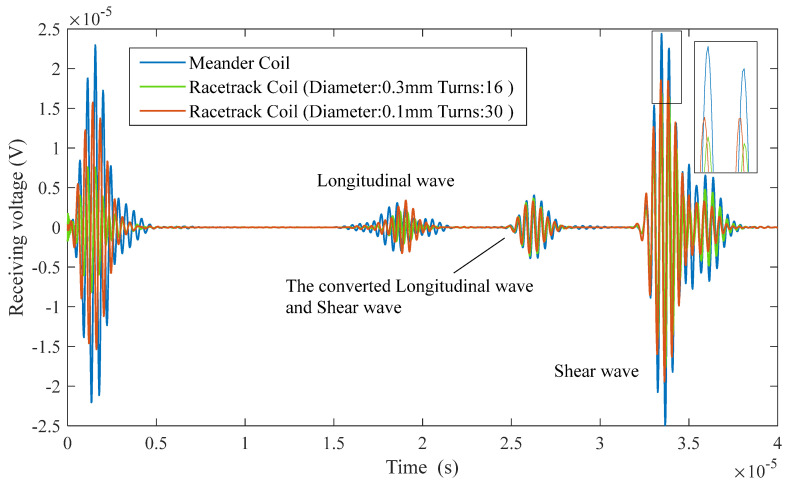
The receiving voltage signal of different coils.

Figure 9 presents a comparison of the received signal between the single-coil probe with a meander excited coil and the dual coil probe with a racetrack received coil. This study found that the type of received coil did not affect the overall shape of the time-domain waveform of the received signal. Based on the characteristic that the speed of L-wave is twice that of SVs, the first echo signal in the figure corresponds to the reflected echo of the L-wave, while the second echo signal is the result of the superposition of echoes generated by the longitudinal-to-shear and shear-to-longitudinal mode conversions. The final echo signal, which has a higher intensity peak, was confirmed through analysis to be the effective reflection of obliquely incident SVs.

Quantitative analysis shows that the maximum voltage amplitude of the dual coil with a wire diameter of 0.3 mm and 16 turns is 1.7 × 10^−6^ V; for the dual coil with a wire diameter of 0.1 mm and 30 turns, this maximum value is 1.85 × 10^−6^ V. Meanwhile, the maximum value of the single-turn meander coil reaches 2.45 × 10^−6^ V. The voltage amplitude of the effective signal received by the dual coil is approximately 75% of that received by the single coil. This difference can be attributed to several factors. First, the single coil uses the same coil for both transmission and reception, which maximizes the propagation direction of the ultrasound and the efficiency of energy transfer, enhancing the electromagnetic coupling effect of the detection signal, thereby increasing the amplitude of the echo signal. In contrast, in the dual coil, the transmission and reception coils are separate, and there is a certain spatial distance and relative positioning difference between the excited and received coils, resulting in a reduction in the amplitude of the echo signal received by the racetrack coil. Finally, considering that practical conditions are not ideal (such as the resistance of the coils), for the one-excitation–one-reception racetrack received coil, the separation of the excited and received coils introduces additional external noise interference and electromagnetic coupling between the two coils, further weakening the echo signal.

In summary, considering the critical impact of signal strength on the sensitivity of dynamic rail detection, the single meander coil is ultimately selected as the preferred solution for dynamic rail flaw detection.

## 4. Analysis and Verification of Defect Detection Performance of Optimized EMAT Probes

### 4.1. Characteristics of Received Signals from Different Defects

After the optimization of parameters, both the strength and SNR of the signal have been significantly improved. To comprehensively assess the actual detection capabilities and echo signal characteristics of the optimized probe for various types of defects, two typical artificial defects were selected as detection targets: a horizontal through-hole (diameter 5 mm, depth 50 mm) and a flat-bottom hole (diameter 3 mm, length 25 mm, oriented at 37° relative to the edge of the test block).

**Figure 10:**
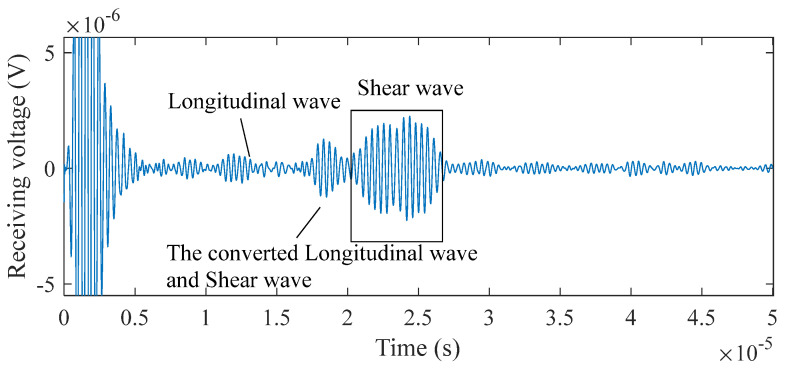
Receiving signals of the flat-bottomed hole.

**Figure 11:**
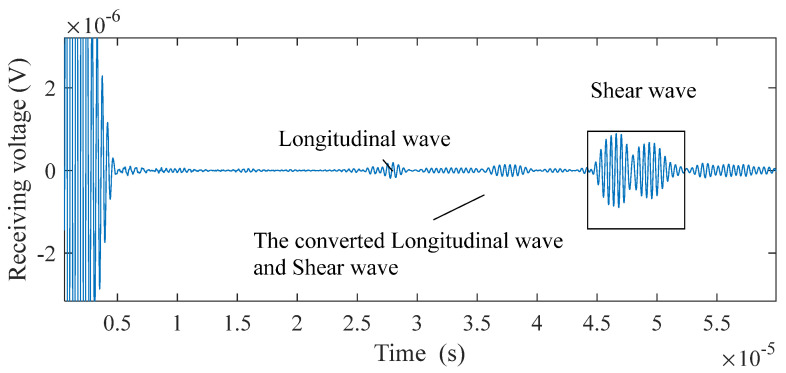
Receiving signals of the horizontal through−hole.

As illustrated in Figure 10 and Figure 11, the probe detects signals corresponding to two distinct types of defects. The echo signal associated with each defect comprises L-waves, converted waves consisting of both L-waves and SVs, as well as SVs themselves. Two peaks characterize each SV. This phenomenon occurs because voltage is induced across the coil only when particle vibrations are suddenly generated beneath it: the first peak corresponds to the echo returning directly under the coil, while the second arises as the wave continues to propagate along the surface. This effect is particularly pronounced due to the greater intensity of SVs compared to that of other wave types. For the flat-bottomed hole, the maximum intensity of the SV signal is 2.26 × 10^−6^ V, while that of the converted wave and L-wave is 1.26 × 10^−6^ V and 6.28 × 10^−6^ V, respectively. For the horizontal through-hole, the maximum intensity of the SV signal is 8.89 × 10^−7^ V, compared to 1.37 × 10^−7^ V for the converted wave and 1.86 × 10^−7^ V for the L-wave, respectively.

The signal strength of flat-bottomed holes exceeds that of horizontal through-holes. As SVs serve as the primary pulse, only SV echo signals are regarded as effective waves, whereas L-waves and converted waves are classified as interference requiring filtration. In steel, the propagation speed of SVs is half that of L-waves. Thus, time-gating can be applied to filter interference: L-waves and converted waves are removed by setting windows at 0.5 and 0.75 times the arrival time of the strongest SV echo, respectively. After filtering these interfering waves, the SNR of a 5 mm diameter, 50 mm deep horizontal through-hole detected by the probe reaches 14.3 dB. For a flat-bottomed hole with dimensions of 3 mm in diameter, 25 mm in length, and 50 mm in depth, the probe achieves an SNR of 14.0 dB.

### 4.2. Evaluation of Detection Capability for Rail Horizontal-Through-Hole

**Figure 12:**
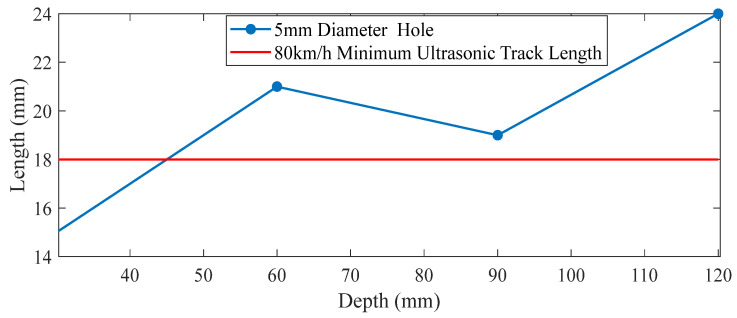
The simulation results of different detection scan distances.

As described in Section 3.2, the detection scan distance, the distance between the initial position and the end position where the defect can be detected (Δ*l*), plays a critical role in determining whether defects can be identified. At the same repetition frequency, a higher detection speed results in a greater distance between measurement points, which in turn affects the required detection scan distance. Taking a 5 mm diameter transverse through-hole as an example, simulations were conducted to calculate the detection scan distances at burial depths of 30 mm, 60 mm, 90 mm, and 120 mm, respectively. The simulation results are presented in Figure 12.

It can be observed from the figure that as the depth of the transverse hole increases, the detection scan distance exhibits an “N”-shaped trend. The shortest detection scan distance occurs when the defect depth is approximately 90 mm. When the depth exceeds 90 mm, the detection scan distance increases monotonically with depth. This phenomenon is primarily attributed to the increased emission angle. Generally, the maximum inspection speed of a flaw detection vehicle is 80 km/h, with a pulse transmission frequency ranging from 3.5 kHz to 4 kHz, which varies slightly with changes in speed. Assuming a transmission frequency of 3.7 kHz and a traveling speed of 80 km/h (equivalent to 22.22 m/s), the scanning interval can be calculated as 22.22 m/s/3700 Hz = 6 mm. Since the system requires at least three B-Scan echo points for reliable defect identification, the probe detection scan distance is set at 18 mm under the maximum scanning interval of 6 mm. Therefore, at a detection speed of 80 km/h, a 5 mm diameter transverse hole located at a depth between 0 mm and 47 mm cannot be reliably detected, and the detection speed must be reduced to ensure sufficient measurement resolution.

## 5. Conclusions

In response to the requirements of rapid rail detection and the developmental characteristics of rail fatigue damage, this study compared three core performance indicators—emission angle, detection scan distance, and intensity—between LFSV-EMAT and USV-EMAT. The USV-EMAT was selected for rapid rail detection, given its better performance in the compared indicators. Additionally, the structural parameters of the probe were optimized using orthogonal experiment theory. The following conclusions are drawn:

1. Although SV intensity generated by LFSV-EMAT is slightly higher than that of USV-EMAT, its wider emission angle and longer detection scan distance make it more advantageous for application in dynamic rail detection.
2. The most significant factor influencing acoustic wave directivity is the number of coil turns. Regarding echo intensity, the lift-off distance has the most significant effect. For the detection blind zone, the number of coil turns has the greatest impact. And the voltage amplitude of the effective signal received by the one-excitation–one-reception racetrack coil is weaker than that received by the spontaneous excitation–reception meander coil.
3. The signal-to-noise ratio (SNR) of a 5 mm diameter, 50 mm deep horizontal through-hole detected by the optimized probe reaches 14.3 dB. For a flat-bottomed hole with dimensions of 3 mm in diameter, 25 mm in length, and 50 mm in depth, the probe achieves an SNR of 14.0 dB.
4. As the depth of the transverse hole increases, the detection scan distance exhibits an “N”-shaped trend. The shortest detection scan distance occurs when the defect depth is approximately 90 mm.

While the non-contact unidirectional USV-EMAT with a larger emission angle exhibits greater suitability for rapid rail flaw detection, it suffers from lower transduction efficiency compared to EMATs with a traditional structure. Therefore, in subsequent research, we will prioritize improving its transduction efficiency and carry out systematic experimental verification of its anti-vibration performance to promote its practical application in dynamic rail inspection scenarios.

## Figures and Tables

**Table 1:** EAMT simulation model parameters.

Object	Parameter	Value
Coil	Width	0.2 mm
	Height	0.04 mm
	Lift-off	0.2 mm
Permanent Magnet	Size	30 mm × 15 mm
	Lift-off	1.2 mm
Rail	Young’s Modulus	2.06 × 10^11^ Pa
	Rail Poisson’s Ratio	0.3
	Rail Density	7850 kg/m^3^

**Table 2:** Influencing factors and their corresponding levels of orthogonal experiment.

Level	Turns	Height (mm)	Width (mm)	Lift-Off (mm)
1	8	0.04	0.1	0.2
2	10	0.08	0.2	0.4
3	12	0.12	0.3	0.6
4	14	0.16	0.4	0.8
5	16	0.20	0.5	1.0

**Table 3:** Orthogonal experimental table for parameter optimization of EMAT.

Run	Turns	Height (mm)	Width (mm)	Lift-Off (mm)	Directivity (°)	Strength (mV)	Blind Zone (mm)
1	8	0.04	0.1	0.2	35	0.0140	6.195
2	8	0.08	0.2	0.4	35	0.0077	6.275
3	8	0.12	0.3	0.6	35	0.0039	6.354
4	8	0.16	0.4	0.8	34	0.0017	6.402
5	8	0.20	0.5	1.0	34	0.0012	6.449
6	10	0.04	0.2	0.6	36	0.0007	6.621
7	10	0.08	0.3	0.8	36	0.0038	6.157
8	10	0.12	0.4	1.0	36	0.0017	6.184
9	10	0.16	0.5	0.2	36	0.0200	6.556
10	10	0.20	0.1	0.4	36	0.0125	6.837
11	12	0.04	0.3	1.0	37	0.0021	6.902
12	12	0.08	0.4	0.2	37	0.0244	7.539
13	12	0.12	0.5	0.4	37	0.0142	7.574
14	12	0.16	0.1	0.6	37	0.0084	7.577
15	12	0.20	0.2	0.8	37	0.0043	6.958
16	14	0.04	0.4	0.4	38	0.0136	7.457
17	14	0.08	0.5	0.6	38	0.0079	7.503
18	14	0.12	0.1	0.8	38	0.0043	7.355
19	14	0.16	0.2	1.0	38	0.0020	7.307
20	14	0.20	0.3	0.2	38	0.0220	8.221
21	16	0.04	0.5	0.8	38	0.0046	8.163
22	16	0.08	0.1	1.0	39	0.0022	7.434
23	16	0.12	0.2	0.2	38	0.0260	8.784
24	16	0.16	0.3	0.4	38	0.0154	8.112
25	16	0.20	0.4	0.6	38	0.0085	8.143

**Table 4:** Analysis of the orthogonal experimental results for parameter optimization of EMAT’s SV wave detection coil.

Test Results	Level	Design Factors			
		Turns	Height	Width	Lift-Off
Directivity (°)	1	**34.6**	36.8	**37**	36.8
	2	36.0	**37**	36.8	36.8
	3	37.0	36.8	36.8	36.8
	4	38.0	**36.6**	36.6	**36.6**
	5	**38.2**	36.6	**36.6**	**36.8**
	*T*_1_	3.6	0.4	0.4	0.2
	Influence rank	Turns (3.6) > Height (0.4) ≈ Width (0.4) > Lift-off (0.2)			
Blind zone (mm)	1	**6.335**	7.068	**7.080**	**7.179**
	2	6.471	**6.986**	7.101	7.059
	3	7.310	7.250	7.149	7.120
	4	7.569	7.191	7.145	**6.883**
	5	**8.127**	**7.322**	**7.249**	6.935
	*T*_2_	1.792	0.336	0.169	0.296
	Influence rank	Turns (1.79) > Height (0.336) > Lift-off (0.296) > Width (0.169)			
Echo strength (mV)	1	**0.0057**	**0.0070**	0.0083	**0.0217**
	2	0.0077	0.0092	**0.0081**	0.0127
	3	0.0099	0.0096	**0.0098**	0.0059
	4	0.0092	0.0095	0.0096	0.0037
	5	**0.0113**	**0.0097**	0.0096	**0.0018**
	*T*_3_	0.0056	0.0027	0.0017	0.0199
	Influence rank	Lift-off (0.0199) > Turns (0.0056) > Height (0.0027) > Width (0.0017)			

## Data Availability

Data underlying the results presented in this paper are not publicly available at this time but may be obtained from the author upon reasonable request.
